# Discrete Entity Analysis via Microwave-Induced Nitrogen Plasma–Mass Spectrometry in Single-Event Mode

**DOI:** 10.1021/acs.analchem.5c04341

**Published:** 2025-10-14

**Authors:** Ana Rua-Ibarz, Flávio V. Nakadi, Eduardo Bolea-Fernandez, Antonio Bazo, Beatrice Battistella, Anna Matiushkina, Ute Resch-Genger, Carlos Abad, Martín Resano

**Affiliations:** † Department of Analytical Chemistry, Aragon Institute of Engineering Research (I3A), 16765 University of Zaragoza, Zaragoza 50009, Spain; ‡ 42220 Bundesanstalt für Materialforschung und -prüfung (BAM), Richard-Willstätter-Str. 11, D-12489 Berlin, Germany

## Abstract

In this work, single-event microwave-induced nitrogen plasma–mass spectrometry (single-event MINP-MS) was evaluated for the first time for the analysis of discrete entities such as nanoparticles, biological cells, and microplastics. Nitrogen (N_2_) effectively overcomes Ar-based polyatomic interferences, enabling (ultra)­trace element determination of Fe and Se using their most abundant isotopes, ^56^Fe (91.66%) and ^80^Se (49.82%). Iron oxide nanoparticles (Fe_2_O_3_ NPs) ranging from 20 to 70 nm were accurately characterized, with excellent agreement with established sizing techniques, such as transmission electron microscopy (TEM) and dynamic light scattering (DLS). A limit of detection (LoD) of 8.6 ag for Fe—equivalent to an LoD_size_ of 19 nm for Fe_2_O_3_—was achieved, which is significantly lower than recent values reported for high-end quadrupole-based ICP-MS. Selenium nanoparticles (SeNPs) of 150 and 250 nm were also accurately characterized, without the N_2_-based plasma experiencing issues handling relatively large metallic NPs (linearity, *R* ^2^ = 0.9994). Se-enriched yeast cells (SELM-1 certified reference material) were successfully analyzed via single-cell MINP-MS using external calibration based on SeNPs and a transport efficiency-independent approach. In addition, 2–3 μm polystyrene (PS) and polytetrafluoroethylene (PTFE) were accurately sized by monitoring ^12^C^+^, confirming the method’s suitability for handling micrometer-sized polymeric materials (microplastics). The average duration of individual events (680 ± 160 μs) suggests that the digestion of individual entities in N_2_-based plasmas is comparable to that in Ar-based plasmas. These results open new avenues for this instrumentation as an alternative to ICP ionization sources, also in the context of discrete entity analysis.

## Graphical abstract

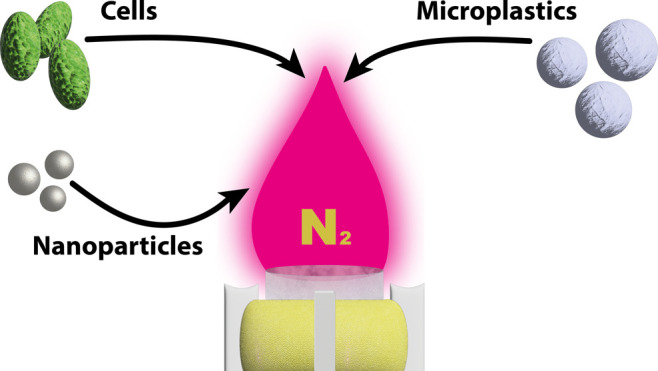

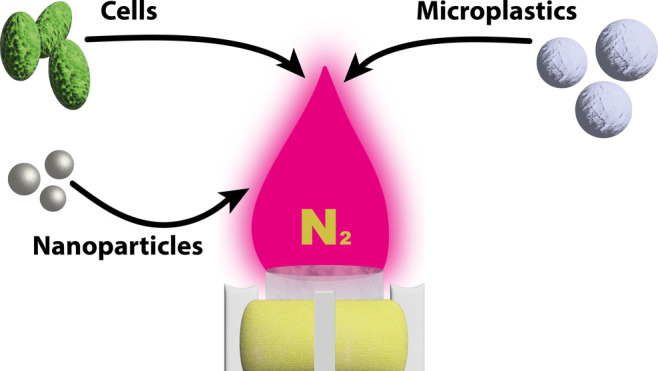

## Introduction

Inductively coupled plasma-mass spectrometry (ICP-MS) is among the most powerful techniques for (ultra-) trace elemental analysis.^1^ However, it suffers from several drawbacks, with the occurrence of spectral interferences – especially in the low mass range (<81 amu) – being one of the most significant. The entire evolution of ICP-MS instrumentation can largely be seen as a series of developments aimed at providing users with new tools to overcome these interferences.^2^ These include the introduction of collision/reaction cell (CRC) technology in quadrupole-based ICP-MS, high-resolution sector-field ICP-MS, and, more recently, tandem ICP-MS instrumentation.^3–6^ This range of instrumental technologies relies on chemical or physical resolutions to overcome spectral overlap.^7^

Among these interferences, the occurrence of argon-based (poly)­atomic ions is particularly important, as argon (Ar) flows >15 L min^–1^ are typically required to sustain the plasma, making Ar and its species ubiquitous in ICP-MS analyses. To address this, the introduction of nitrogen (N_2_) into an Ar plasma – forming a mixed Ar–N_2_ plasma – has been shown to reduce Ar-based polyatomic interferences, although this approach has been associated with a slight increase in detection limits (LoD).^8^ Additionally, Ar consumption substantially contributes to the high cost of routine ICP-MS operation. In recent years, low-flow plasma torches have been developed to reduce Ar gas consumption. However, this approach brings about specific challenges, such as a lower-energy plasma that may compromise the detection of elements with high ionization energies.^9–11^

Recently, the MICAP source (microwave-sustained inductively coupled atmospheric-pressure plasma) has been introduced as a cost-effective alternative to Ar-based plasmas. In this study, microwave-induced nitrogen plasma (MINP) is adopted as the generic term for this technology. Initially implemented with optical emission spectrometry (MINP-OES), it proved successful for analyzing complex matrix samples.^12–15^ This prompted an early coupling with mass spectrometry (MINP-MS), aiming to explore its broader potential. Nitrogen (N_2_) is not only significantly less expensive than Ar – reducing operating costs by 40–60% at comparable flow rates – but also more readily available. Additionally, its use addresses the occurrence of Ar-based isobaric and polyatomic interferences. Despite these advantages, the technique remains largely unexplored, and further research is still required to fully evaluate the potential of N_2_ as a viable alternative to Ar-based plasmas. Currently, only a few prototypes are in use by the research groups of Dr. Günther at ETH Zurich and Dr. Abad at BAM.^16–22^ The MINP source has also been coupled to time-of-flight (ToF) and quadrupole (Q) mass analyzers; however, existing studies focused on bulk, speciation, and laser ablation analyses. Very recently (2025), a first MINP-ToF-MS instrument was commercialized by Tofwerk AG (as mipTOF).^23^ This new instrument is intended for use as a portable, field-deployable system for real-time quantification of trace elements and metals in air.

Despite the growing scientific interest in this technology, the suitability of the MINP-MS technique for use in one of ICP-MS’s current key features – single-event analysis – has not yet been evaluated.^24^ The single-event mode is based on the one-by-one introduction of individual entities into the plasma, where they are individually ionized. The resulting ion cloud is introduced into the mass analyzer for subsequent detection, yielding a short transient ion signal (≈500 μs).^25,26^ With appropriate calibration, the signal intensities of these individual events can be converted into the analyte mass distribution and, under a number of assumptions, into a size distribution.^27,28^ This mode is widely used in ICP-MS for characterizing nanoparticles (NPs), biological cells, and even microplastics (MPs).^29–31^ However, the prototype nature of MINP-MS systems has likely limited their use in single-event mode, which requires the monitoring of ultrafast transient signals in time-resolved analysis, and the subsequent dedicated data processing strategy.

In this work, the applicability of microwave-induced nitrogen plasma-mass spectrometry operated in single-event mode (single-event MINP-MS) has been evaluated for the analysis of NPs, cells, and MPs. The potential of the technique for individually detecting these small entities with a high sensitivity, free from spectral interference, has been assessed. Special attention has been paid to elements that are heavily interfered in an Ar-based plasma, such as Fe and Se, as their most abundant isotopes, 56 and 80 amu, overlap with ArO^+^ and Ar_2_^+^ signals. The achievable figures of merit for NP characterization and cell analysis – applications where these elements are particularly relevant – have been evaluated, along with the suitability of an N_2_-based plasma for analyzing relatively large and robust particulate materials, such as MPs, in single-event mode. This can pave the road to many future applications of this emerging technique and instrumentation in the life and material sciences.

## Materials and Methods

### Instrumentation

All measurements were carried out using a microwave-induced nitrogen plasma source (MINP) coupled to a single quadrupole mass spectrometer (PlasmaQuant MS Elite, Analytik Jena GmbH, Germany). The mass spectrometer and all coupling/interface hardware and software were supplied and installed by Analytik Jena. Details of the operating principle and performance of the setup are provided elsewhere.^17,18,21^ The sample introduction system comprises a concentric pneumatic nebulizer (MicroMist, USA) and a cooled Scott-type double-pass spray chamber (3 °C). The sample uptake rate was found to be between 300 and 400 μL min^–1^. Nitrogen 5.0 (N_2_ purity ≥ 99.999%, Linde AG, Germany) was used as the general nebulizer, auxiliary, and plasma gas for most MINP-MS measurements. Furthermore, N_2_ 6.0 (N_2_ purity ≥ 99.9999%, Linde AG) was used to assess the effect of N_2_ purity on the performance of specific analyses. Argon 5.0 (Ar purity ≥ 99.999%, Linde AG) was used to ignite the plasma. Ignition was initiated by a spark assisted with a 3–5 s Ar flow at 1.5 L min^–1^, which helped propagate the discharge into the dielectric-resonator region. Immediately after ignition, all gas flows were switched to N_2_. The instrument was tuned daily across the full mass range using an ICP-MS IS solution (Analytik Jena GmbH) containing 5 μg L^–^^1^ of ^6^Li, ^45^Sc, ^89^Y, ^115^In, ^159^Tb, and ^209^Bi in 0.28 M HNO_3_ to ensure optimum operating conditions. The Aspect MS software (Analytik Jena GmbH) was used to control the instrument and acquire data. Single-event monitoring was performed with a dwell time of 100 μs and a variable settling time of ≈30 μs, which was recorded for each dwell. During data processing, the counts acquired per dwell time were multiplied by the exact factor accounting for the corresponding settling period, ensuring that results reflected the full duty cycle to avoid bias. The raw data were exported as .csv files. Instrument settings and data acquisition parameters are listed in Table 1. Transmission electron microscopy (TEM) measurements were performed using a Talos F200S microscope (Thermo Fisher Scientific, Germany). Dynamic light scattering (DLS) measurements were carried out at 25 °C with a Zetasizer Nano ZS (Malvern Panalytical Ltd., UK). The conditions for the DLS and TEM measurements are provided in the Supporting Information (SI).

**Table 1:** MINP-MS Operating Conditions

measurement parameters	
plasma power	1500 W
nebulizer gas flow	1.25 L min^–1^
auxiliary gas flow	2.25 L min^–1^
plasma gas flow	9.00 L min^–1^
sampling depth	5.0 mm
sampling cone	Pt 1.1 mm
skimmer cone	Ni 0.5 mm

### Reagents and Standards

All reagents used were of analytical purity. Ultrapure water (18.2 MΩ cm) was obtained from a Milli-Q water purification system (Merck Millipore, Germany). High-purity 14 M HNO_3_ and 12 M HCl were obtained from Merck (Germany). For method development and calibration purposes for NP characterization, 1000 mg L^–1^ ICP stock solutions of Au, Fe, and Se (Merck) were appropriately diluted in 0.24 M HCl (Au) and 0.28 M HNO_3_ (Fe and Se). For calibration when aiming at polymer-based microparticle analysis, a carbon-containing solution was prepared by dissolving approximately 150 mg of citric acid (Merck) in ultrapure water. The standard solutions contained 0, 1.0, 2.0, 3.0, 4.0, and 5.0 μg L^–1^ of Au, Fe, and Se, and 0, 56, 112, and 224 μg L^–1^ of C.

Suspensions of spherical 40, 60, and 70 nm AuNPs (HiQ-Nano, Italy) were used to determine the ionic transport efficiency (TE) based on the particle size method.^26^ This approach was used for sizing both Fe- and Se-based NPs as well as polymer-based microparticles.

The performance of the N_2_-based plasma for NPs characterization was evaluated using Fe- and Se-based NPs. For this purpose, the following NPs were analyzed in this work: (i) in-house synthesized 65 nm citrate-coated α-Fe_2_O_3_ NPs,^32^ (ii) magnetic γ-Fe_2_O_3_ NPs (FeraSpin XL and XXL, nanoPET, Germany) designed for magnetic particle imaging (MPI), and (iii) SeNPs from Merck with average sizes of 150 nm (range: 140–160 nm) and 236 nm (range: 230–270 nm). All NP suspensions were prepared with particle number concentrations of approximately 5 × 10^5^ particles mL^–1^. Details on the synthesis of α-Fe_2_O_3_ NPs are provided in the SI.

As biological entities, the SELM-1 selenium-enriched yeast certified reference material (CRM) from the National Research Council Canada (NRC) was analyzed. The SELM-1 provides information on the total selenium, selenomethionine, and methionine contents, while the mass of selenium per individual cell was previously determined via single-cell ICP-MS (SC-ICP-MS).^33,34^ Single-cell MINP-MS-focused analysis (SC-MINP-MS) relied on the external calibration approach using NPs composed of the target analyte: 150 and 250 nm SeNPs. Approximately 20 mg of the SELM-1 yeast CRM was suspended in 10 mL of ultrapure water, and vortexed for at least 1 min prior to analysis, following the procedure described in previous works to enable comparison of results.^33,34^ The cell suspension was further diluted (20-fold) in ultrapure water to reach a cell density of approximately 1.5 × 10^6^ cells mL^–1^.

The analysis of polymeric microparticles (MPs) was performed using two 2.5 μm polystyrene microparticle standards doped with 4 and 6 lanthanide elements, respectively (PS; Standard Biotools, USA) and a 3 μm polytetrafluoroethylene microparticle standard (PTFE; Polysciences Inc., USA). The PS microparticle standards were purchased as a particle suspension (3.30 × 10^5^ particles mL^–1^) and were diluted by a factor of 2, yielding a concentration of 1.75 × 10^5^ particles mL^–1^. The PTFE microparticle standard was purchased as a solid material and was suspended in a 0.1% m v^–1^ Triton X-100 solution (Merck) prior to dilution to a concentration of 1.0 × 10^6^ particles mL^–1^.

All discrete entities – NPs, cells, and MPs – were measured in triplicate (*n* = 3). The results presented correspond to the cumulative distribution, obtained by pooling the data from the three measurement replicates for each entity type.

### Data Processing

The Hyper Dimensional Image Processing software (HDIP v1.8.4) was used for processing the data exported from the Aspect MS software (Analytik Jena GmbH). HDIP enabled the identification and integration of the individual events, as described elsewhere.^35–37^ The duration of individual events (i.e., the width of the peaks corresponding to single entities) was determined using an in-house developed script, as described elsewhere.^28^ For further data processing and graphics preparation, OriginPro software (version 2021b, 9.85 OriginLab Corporation, USA) was used. This software was also used to obtain the central values and standard deviations (SD) from Gaussian fitting distributions, as well as to deconvolute the true individual-level distribution in cases involving double events or aggregates (single-cell results).

## Results and Discussion

### Characterization of Iron Oxide and Selenium Nanoparticles

To assess the suitability of the N_2_-based plasma for the analysis of NPs in single-particle mode (SP-MINP-MS), Fe- and Se-based NPs were studied first. As discussed before, these NPs were selected because both Fe and Se signals are strongly affected by Ar-based polyatomic interferences (e.g., ArO^+^ and Ar_2_^+^, respectively) in conventional Ar-based plasmas. ICP-MS measurements often rely on chemical or physical resolution approaches to overcome such spectral overlap.^2^ However, N_2_ plasmas are affected by different types of spectral interferences, potentially allowing for the interference-free determination of those elements the quantification of which is traditionally hampered by the overlap with Ar-based polyatomic ions.^8^

For the characterization of Fe-based NPs, the figures of merit of the MINP-MS for the determination of ^56^Fe^+^ were first evaluated. The sensitivity was determined to be approximately 250,000 counts per second (cps) L μg^–1^. This high sensitivity can be attributed to the ability of monitoring the most abundant Fe isotope (56 amu), while the preferential transmission of relatively light elements in an N_2_ plasma and the absence of an intense Ar ion beam at the mass-to-charge (*m*/*z*) ratio of the target nuclide can significantly contribute to an improved ion transmission efficiency, and thus, a higher sensitivity.^16,38^ However, the measurement of a blank solution (0.14 M HNO_3_) yielded an intensity of approximately 10,000 cps. This relatively high blank signal might be attributed to the occurrence of N_2_-based polyatomic interferences (e.g., N_4_^+^ at *m*/*z* = 56).^16^ However, additional measurements resulted in background signals of approximately 500 cps, indicating minimal impact of this interface on the detected ^56^Fe^+^ intensity and pointing instead to Fe contamination at ultratrace levels as the most likely explanation. In any case, it should be noted that this background intensity becomes less relevant when monitoring ultrafast transient signals in single-event mode (this background signal corresponds to only 1 count with a 100 μs dwell time).

For method development in the context of Fe-based NP sizing, a well-characterized 65 nm Fe_2_O_3_ NPs suspension was analyzed. Figure 1A,B show the time-resolved signal and average event duration, and the SP-MINP-MS size distribution results, as compared to those obtained via TEM. As can be seen, a very good agreement was found between both techniques (66 ± 10 and 68 ± 19 nm for TEM and SP-MINP-MS, respectively). To further assess the potential of the method under more challenging conditions, smaller Fe-based NPs were also analyzed. In this case, the size distributions of the commercial MRI contrast agents FeraSpin XL and FeraSpin XXL suspensions, consisting of Fe_2_O_3_ NPs previously characterized by DLS and TEM, were obtained using SP-MINP-MS. The results are shown in Figure 1C,D. TEM analysis did not allow for a suitable characterization of the relatively polydisperse FeraSpin XL and FeraSpin XXL Fe_2_O_3_ NPs, as only aggregates of smaller NPs could be visualized in the dry material, and it is very challenging to estimate the actual size of the NPs in suspension. However, a good agreement was observed between the SP-MINP-MS and DLS results for FeraSpin XL and FeraSpin XXL with values of 28 ± 6 and 30 ± 6 nm, and 29 ± 9 and 36 ± 9 nm for SP-MINP-MS and DLS, respectively, obtained upon measurement of the NP suspensions. This confirms the potential of SP-MINP-MS for NP sizing. The high sensitivity of the MINP-MS instrument for Fe enabled the accurate characterization of Fe-based NPs with sizes <30 nm, while in previous works, high-end ICP-MS instrumentation faced difficulties in characterizing Fe-based NPs with sizes below 50 nm.^7,35^ This was further confirmed by calculating an LoD_mass_ of 8.6 ag, corresponding to an LoD_size_ of 19 nm, for Fe_2_O_3_ NPs.

**Figure 1:**
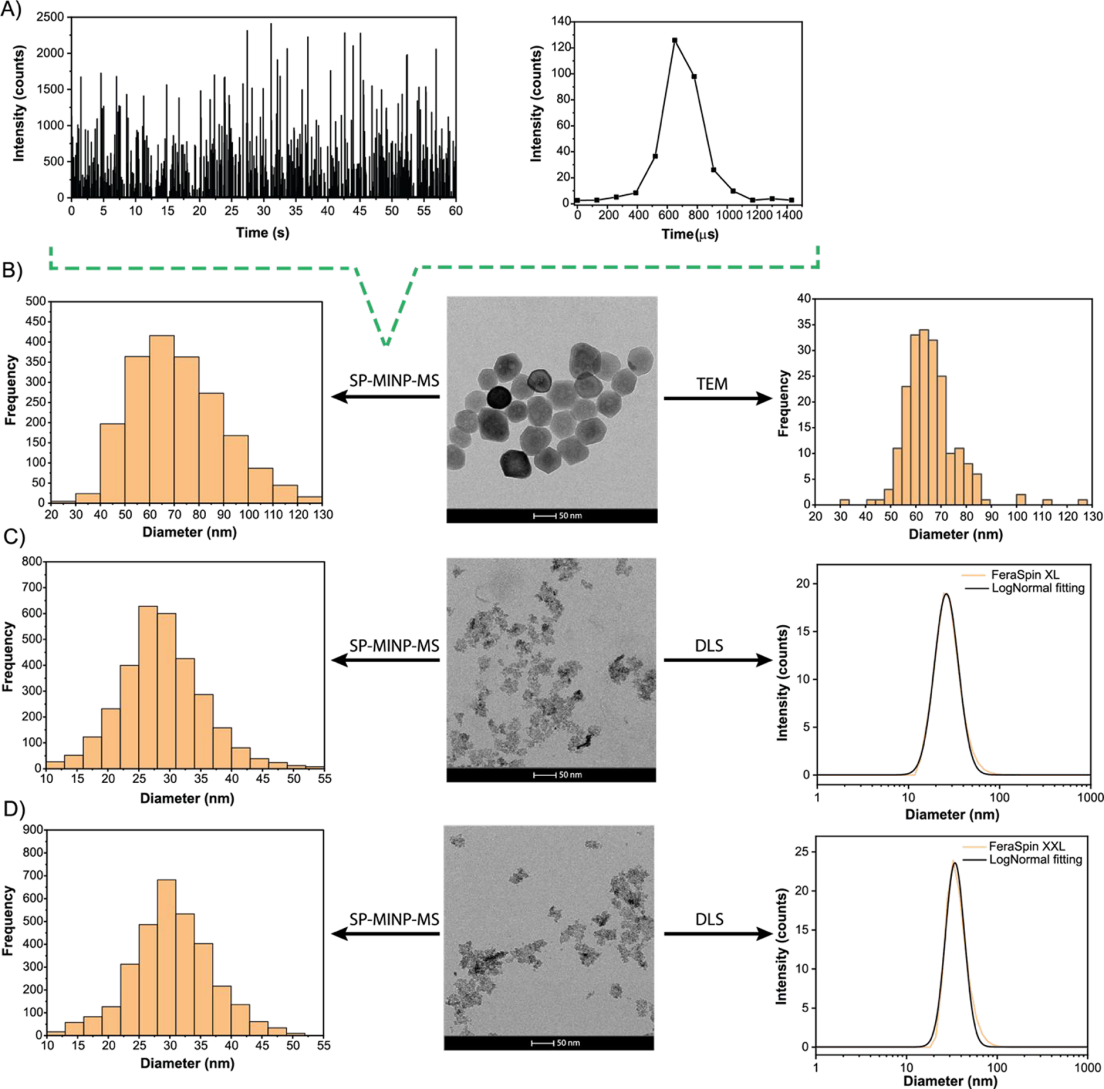
Characterization of different Fe_2_O_3_ NPs. (A) Transient SP-MINP-MS signal, with an example of an event duration of the 65 nm Fe_2_O_3_ NP standard suspension. (B) SP-MINP-MS-derived size distribution, TEM images, and TEM-derived size distribution of the 65 nm Fe_2_O_3_ NP suspension. (C) SP-MINP-MS-derived distribution, TEM images, and DLS-derived size distribution of the FeraSpin XL NP suspension. (D) SP-MINP-MS-derived distribution, TEM images, and DLS-derived size distribution of the FeraSpin XXL NP suspension.

Characterization of NPs via SP-MINP-MS was further explored by using a completely different NP type: metallic SeNPs. An interference-free methodology was developed by relying on the N_2_ plasma, thus avoiding the Ar dimer signal, which overlaps with the most abundant Se isotope (^80^Se). The sensitivity was found to be 1,200 cps L μg^–1^, which can be considered moderate when compared to that of Fe. This difference is mainly due to the high ionization potential of Se (9.75 eV) compared to that of Fe (7.9 eV). However, with more specific tuning of the instrument for this particular *m*/*z* ratio, rather than general tuning across the full mass range, as done here, improved figures of merit for the determination of Se are likely. To evaluate the ability of the N_2_ plasma to handle metallic NPs that are, a priori, more difficult to fully digest, SeNPs of larger sizes than those used for Fe were selected: 150 and 250 nm. Figure 2A,B show the results obtained for characterizing both SeNP sizes via SP-MINP-MS, compared to the TEM images. The size distributions measured by SP-MINP-MS (165 ± 38 and 248 ± 55 nm; most frequent size = 150 and 237 nm) and TEM (150 ± 5.0 and 240 ± 11 nm) were in very good agreement with the values provided by the SeNP manufacturer. The excellent linearity (*R* ^2^ = 0.9994, see Figure 2C) confirmed the complete digestion of relatively large NPs composed of an element with a high ionization potential, thus pushing the performance limits of the N_2_-based plasma. Moreover, the calibration curve constructed using SeNPs enables the use of external calibration for characterizing other SeNPs or analyzing other Se-containing entities, such as Se-enriched yeast cells (vide infra).

**Figure 2:**
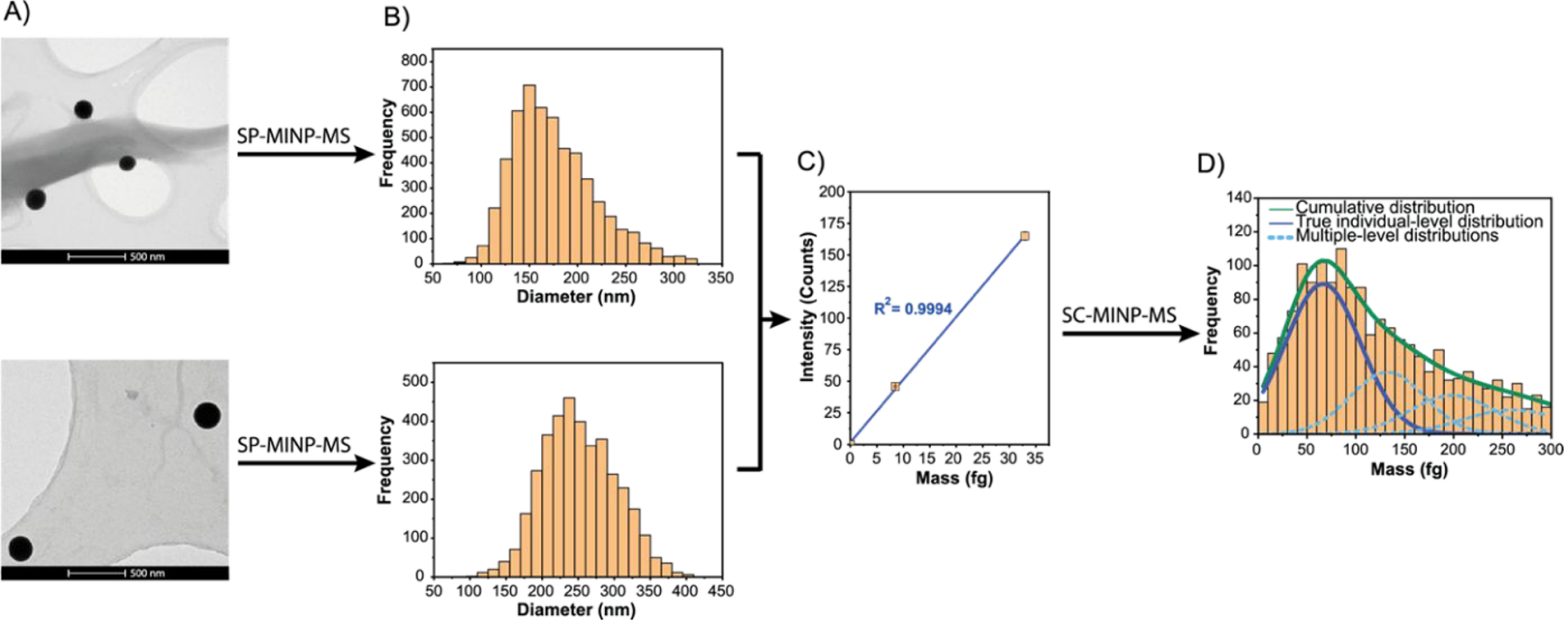
Characterization of SeNPs and analysis of Se-enriched yeast cells. (A) TEM images and (B) SP-MINP-MS-derived size distribution of the 150 and 250 nm SeNP suspensions. (C) Calibration curve constructed using a blank solution and the 150 and 250 nm SeNP suspensions (linearity, *R* ^2^ = 0.9994). (D) SC-MINP-MS-derived mass distribution of Se-enriched yeast cells (SELM-1 certified reference material). Deconvolution enabled the separation of the cumulative distribution (green) into the true individual-level (single-cell) distribution (dark blue) and the higher-level distributions arising from multiple events or cell aggregates (light blue, dashed lines).

Next, in addition to evaluating the accuracy of the results obtained using the MINP-MS operated in single-event mode for characterizing various NP types, the duration of individual events was determined from the baseline peak width. Thereby, potential differences in the digestion of single entities in N_2_-based plasmas compared to Ar-based plasmas were explored. The average event duration for the different NP types ranged from 470 to 870 μs. These values are in good agreement with values previously reported in the literature for ICP-MS analysis.^35,39,40^ The duration of individual events, i.e., the width of the peak corresponding to a single particle, has previously been reported to correlate with NP size.^28,41,42^ In our study, a similar trend was observed, as both Fe and Se NPs exhibited longer event durations for larger particle sizes, e.g., 470 ± 110 and 600 ± 240 μs for Fe_2_O_3_ NPs with sizes of about 30 and 65 nm, and 540 ± 220 μs and 870 ± 330 μs for 150 nm- and 250 nm-sized SeNPs, respectively. These findings confirm that the digestion of individual particles in N_2_-based plasmas is comparable to that in Ar-based plasmas, and that the formation of the ion cloud leads to similar transit times in MINP-MS and ICP-MS systems.

### Analysis of Yeast Cells

Subsequently, to evaluate the potential of the MINP-MS instrument operated in single-event mode for analyzing discrete biological entities, cells were analyzed. For this purpose, the SELM-1 certified reference material, consisting of Se-enriched yeast cells, was used. The mass of Se per individual cell was determined using an external calibration with commercially available SeNP standards (see Figure 2C). This approach offers a straightforward “entity-to-entity” calibration strategy that does not rely on ionic standard solutions and, therefore, avoids the need to calculate a TE, as reported in previous works.^28,34^ A calibration curve was constructed by monitoring the 150 and 250 nm SeNPs along with a blank solution (linearity, *R* ^2^ = 0.9994). The integrated signal intensities obtained from monitoring Se in yeast cells were interpolated onto this calibration curve. Figure 2D shows the SC-MINP-MS results for the analysis of a yeast suspension.

The signal duration of the cell events was determined to be 920 ± 240 μs. This value is not significantly different from that obtained via single-cell analysis in Ar-based plasmas. A comparison of the most frequent Se mass per cell (65 fg cell^–1^) demonstrates an excellent agreement with the results obtained in a previous work using SC-ICP-MS.^34^ The relatively broad distribution can potentially be attributed to the occurrence of cell aggregates. It should be noted that, for this study, a traditional Scott-type double-pass spray chamber, rather than a high-efficiency sample introduction system, was used for introducing all entity types, regardless of their size. This resulted in a significantly lower TE for the cells and required an increase in cell density (from 3.0 × 10^5^ cells mL^–1^ in ref 34 to 1.5 × 10^6^ cells mL^–1^ in the present work) to detect a statistically meaningful number of cell events. Monitoring for longer periods at lower cell density can partly compensate for the low TE of cells, but this approach was hindered by software constraints in ultrafast data collection. The increase in cell density raised the probability of integrating more than one cell’s Se content per event. However, appropriate data treatment enabled the deconvolution of the true individual-level distribution, further confirming the agreement with previous data:^34^ Se mass per cell: 61.7 ± 1.1 and 67 ± 40 fg for SC-ICP-MS and SC-MINP-MS, respectively. It is worth mentioning that the uncertainty in the SC-ICP-MS result refers to the SD of 5 measurement replicates and should not be compared with the SD derived from the Gaussian fit of the SC-MINP-MS distribution. Overall, these results demonstrate the potential of MINP-MS for single-cell analysis.

### Characterization of Polymer-Based Microparticles

Due to increasing concerns about microplastics in the environment and food chain,^43^ the potential of the MINP-MS instrument for analyzing single polymer microparticles was explored. Such polymer-based microparticles are not only significantly larger than NPs but also are generally more rigid and, a priori, more difficult to fully ionize than biological cells, which could present a challenge to N_2_-based plasmas.

In 2020, a pioneering study demonstrated the feasibility of SP-ICP-MS for characterizing MPs with sizes of 1–2.5 μm by relying on their C content (via ^13^C^+^ signal monitoring).^36^ Follow-up studies have shown that Ar-based plasmas are capable of fully digesting polymeric particles up to 20 μm in size.^44–47^

In this work, the potential of MINP-MS for characterizing MPs was evaluated using polystyrene (PS) and polytetrafluoroethylene (PTFE) particles with diameters of 2.5 and 3.0 μm, respectively. PTFE particles possess a higher density than many commonly analyzed polymeric microparticles (2.25 g cm^–3^ for PTFE vs 1.05 g cm^–3^ for PS). Although the Scott-type spray chamber restricted the introduction of larger microparticles, the ability of the MINP to deliver 1.5 kW – comparable to conventional Ar-based ICPs – tentatively suggests a potential for digesting larger polymeric particles, similar to ICP-MS.^48^ The sizes of PS and PTFE particles were determined based on their C content, taking into account prior knowledge of their chemical composition, density, and spherical shape. For this purpose, a calibration curve was constructed using citrate as a C-containing standard (linearity, *R* ^2^ = 0.9997), and the ionic TE was calculated using AuNPs (particle size method). The sensitivity was found to be 9,000 cps L mg^–1^ when monitoring the most abundant C isotope (^12^C^+^). The duration of the individual events was determined to be 630 ± 250, 650 ± 190, and 800 ± 360 μs for 4-lanthanide-doped PS, 6-lanthanide-doped PS, and PTFE microparticles, which, as observed for NPs and cells, did not differ significantly from the values obtained using ICP-MS with Ar plasma. As shown in Figure 3, the SP-MINP-MS size results obtained for the three PS and PTFE microparticle standards were in excellent agreement with the manufacturer’s specifications: 2.67 ± 0.46 and 2.30 ± 0.40 μm (for the 2.5 μm PS) and 2.99 ± 0.61 μm (for the 3.0 μm PTFE). These findings demonstrate that the MINP-MS, when operated in single-event mode, is also capable of characterizing polymer-based microparticles in addition to NPs and cells.

**Figure 3:**
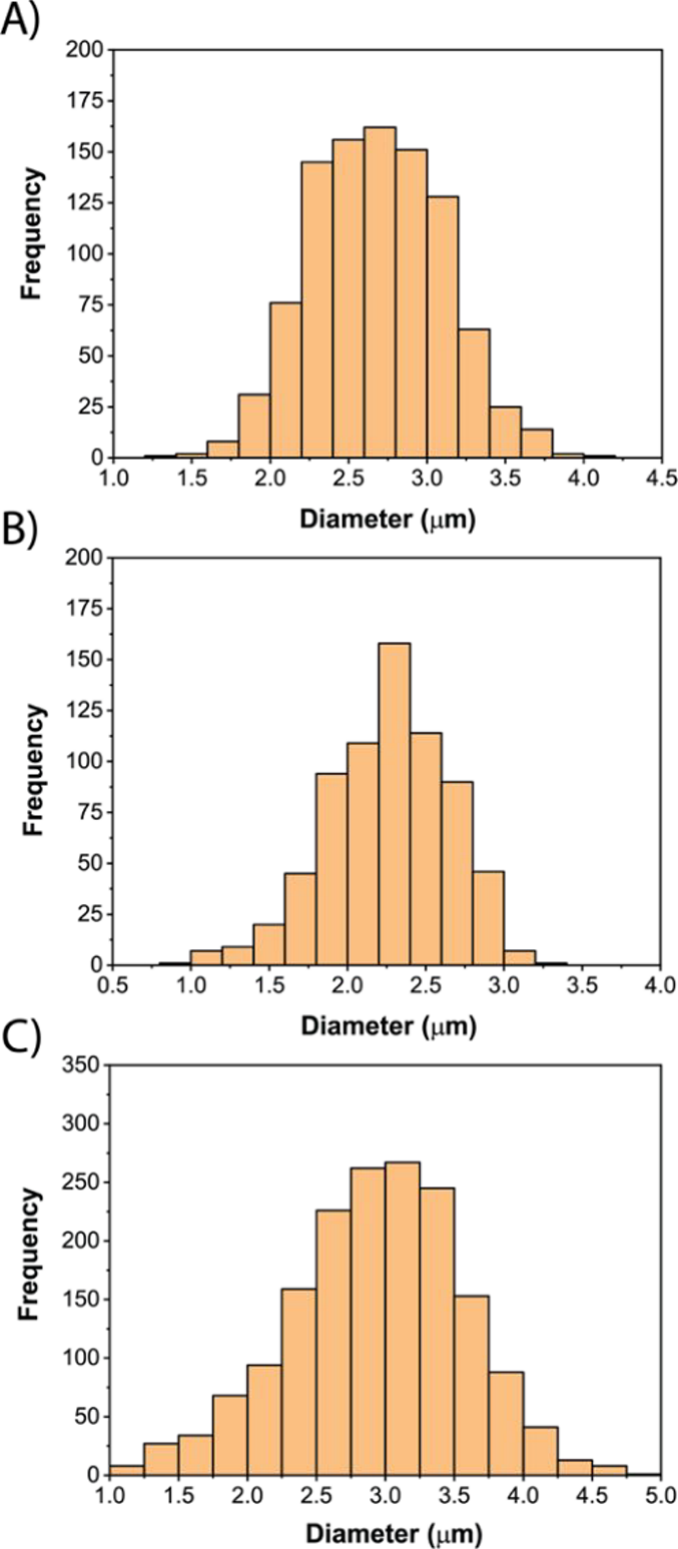
Characterization of polymer-based microparticles (MPs). (A) SP-MINP-MS-derived size distribution of the 2.5 μm PS microparticles doped with 4 lanthanide elements. (B) SP-MINP-MS-derived size distribution of the 2.5 μm PS microparticles doped with 6 lanthanide elements. (C) SP-MINP-MS-derived size distribution of the 3.0 μm PTFE microparticles.

## Conclusions

In this work, the potential of MINP-MS for single-particle and single-cell analysis has been demonstrated. For elements whose determination is traditionally hindered by the occurrence of Ar-based polyatomic interferences in ICP-MS, the N_2_ plasma source used in MINP-MS effectively overcomes this limitation. This has been demonstrated for Fe (Fe_2_O_3_ NPs) and Se (SeNPs and Se-enriched yeast cells), and the setup is likewise expected to improve determinations of other interference-prone elements, such as Ca and K. The excellent sensitivity achievable for Fe and the possibility to determine the most abundant isotope, ^56^Fe, almost free from spectral interferences, enabled a detection limit of <10 ag. This value is, to the best of the authors’ knowledge, lower than any detection limit reported so far using quadrupole-based ICP-MS instrumentation for this element. Beyond NPs and cells, the technique also proved successful in characterizing PS and PTFE microparticles by relying on their C content. The event duration did not differ significantly between ICP-MS and MINP-MS (Ar vs N_2_ plasma), further confirming the latter’s capability to fully digest different types of micro- and nanostructures.

Overall, the N_2_ plasma source succeeded in analyzing all three entity types, independently of their sizes, diverse elemental compositions, or varying densities. For the first time, these results demonstrate that N_2_-plasma-based MINP-MS can serve as a cost-effective alternative to Ar-based plasmas, not only for bulk, speciation, isotopic, and LA analysis, but also as a promising tool for single-entity analysis.

## Supplementary Material



## Notes

Materials and experimental details on the synthesis and characterization of α-Fe_2_O_3_ NPs (PDF)

## References

[1] Van
Acker T., Theiner S., Bolea-Fernandez E., Vanhaecke F., Koellensperger G. (2023). Inductively Coupled Plasma Mass Spectrometry. Nat. Rev. Methods Primers.

[2] Lum T.-S., Leung K. S.-Y. (2016). Strategies to Overcome Spectral Interference
in ICP-MS
Detection. J. Anal. At. Spectrom..

[3] Jakubowski N., Moens L., Vanhaecke F. (1998). Sector Field Mass Spectrometers in
ICP-MS. Spectrochim. Acta, Part B.

[4] Tanner S. D., Baranov V. I., Bandura D. R. (2002). Reaction
Cells and Collision Cells
for ICP-MS: A Tutorial Review. Spectrochim.
Acta, Part B.

[5] Balcaen L., Bolea-Fernandez E., Resano M., Vanhaecke F. (2015). Inductively
Coupled Plasma – Tandem Mass Spectrometry (ICP-MS/MS): A Powerful
and Universal Tool for the Interference-Free Determination of (Ultra)­Trace
Elements – A Tutorial Review. Anal. Chim.
Acta.

[6] Bolea-Fernandez E., Balcaen L., Resano M., Vanhaecke F. (2017). Overcoming
Spectral Overlap via Inductively Coupled Plasma-Tandem Mass Spectrometry
(ICP-MS/MS). A Tutorial Review. J. Anal. At.
Spectrom..

[7] Rua-Ibarz A., Bolea-Fernandez E., Pozo G., Dominguez-Benetton X., Vanhaecke F., Tirez K. (2020). Characterization of Iron Oxide Nanoparticles
by Means of Single-Particle ICP-Mass Spectrometry (SP-ICP-MS) –
Chemical versus Physical Resolution to Overcome Spectral Overlap. J. Anal. At. Spectrom..

[8] Laborda F., Baxter M. J., Crews H. M., Dennis J. (1994). Reduction of Polyatomic
Interferences in Inductively Coupled Plasma Mass Spectrometry by Selection
of Instrumental Parameters and Using an Argon-Nitrogen Plasma: Effect
on Multi-Element Analyses. J. Anal. At. Spectrom..

[9] Evans E. H., Ebdon L. (1991). Comparison of Normal
and Low-Flow Torches for Inductively Coupled
Plasma Mass Spectrometry Using Optimized Operating Conditions. J. Anal. At. Spectrom..

[10] Scheffer A., Brandt R., Engelhard C., Evers S., Jakubowski N., Buscher W. (2006). A New Ion Source Design for Inductively Coupled Plasma
Mass Spectrometry (ICP-MS). J. Anal. At. Spectrom..

[11] Tirk P., Wolfgang M., Wiltsche H. (2016). Reduction
of Argon Consumption to
Less than 2 L min^–1^ by Gas Recycling in Inductively
Coupled Plasma Optical Emission Spectrometry. Anal. Chem..

[12] Wiltsche H., Wolfgang M. (2020). Merits of Microwave Plasmas for Optical Emission Spectrometry
– Characterization of an Axially Viewed Microwave-Sustained,
Inductively Coupled, Atmospheric-Pressure Plasma (MICAP). J. Anal. At. Spectrom..

[13] Wiltsche H., Wolfgang M., Hallwirth F. (2022). Effects of Argon on the Analytical
Properties of a Microwave-Sustained, Inductively Coupled. Atmospheric-Pressure Plasma. J. Anal. At. Spectrom..

[14] Serrano R., Grindlay G., Gras L., Mora J. (2024). Microwave-Sustained
Inductively Coupled Atmospheric-Pressure Plasma (MICAP) for the Elemental
Analysis of Complex Matrix Samples. Talanta.

[15] Pérez-Vázquez J., García-Juan A., Serrano R., Grindlay G., Gras L. (2025). Direct Multi-Elemental
Analysis of Wines by Means of Microwave-Sustained Inductively Coupled
Atmospheric-Pressure Plasma Optical Emission Spectroscopy (MICAP-OES). Microchem. J..

[16] Schild M., Gundlach-Graham A., Menon A., Jevtic J., Pikelja V., Tanner M., Hattendorf B., Günther D. (2018). Replacing
the Argon ICP: Nitrogen Microwave Inductively Coupled Atmospheric-Pressure
Plasma (MICAP) for Mass Spectrometry. Anal.
Chem..

[17] You Z., Akkuş A., Weisheit W., Giray T., Penk S., Buttler S., Recknagel S., Abad C. (2022). Multielement Analysis
in Soils Using Nitrogen Microwave Inductively Coupled Atmospheric-Pressure
Plasma Mass Spectrometry. J. Anal. At. Spectrom..

[18] Winckelmann A., Roik J., Recknagel S., Abad C., You Z. (2023). Investigation
of Matrix Effects in Nitrogen Microwave Inductively Coupled Atmospheric-Pressure
Plasma Mass Spectrometry (MICAP-MS) for Trace Element Analysis in
Steels. J. Anal. At. Spectrom..

[19] Mukta S., Gundlach-Graham A. (2024). Ion Chromatography
– Nitrogen-Sustained Microwave
Inductively Coupled Atmospheric Pressure Plasma – Mass Spectrometry
(IC-MICAP-MS) for Arsenic Speciation Analysis in Rice. J. Anal. At. Spectrom..

[20] Kuonen M., Hattendorf B., Günther D. (2024). Quantification Capabilities of N_2_ MICAP-MS with Solution Nebulization and Aerosol Desolvation. J. Anal. At. Spectrom..

[21] You Z., Winckelmann A., Vogl J., Recknagel S., Abad C. (2024). Determination of Calcium, Iron, and Selenium in Human Serum by Isotope
Dilution Analysis Using Nitrogen Microwave Inductively Coupled Atmospheric
Pressure Plasma Mass Spectrometry (MICAP-MS). Anal. Bioanal. Chem..

[22] Tanen J. L., Jorabchi K. (2025). Nitrogen MICAP with Post-Plasma Ionization
Mass Spectrometry
for Elemental Fluorine Quantitation. J. Anal.
At. Spectrom..

[23] TOFWERK AG. https://www.tofwerk.com/products/miptof/ (last accessed Jul 17, 2025).

[24] Resano M., Aramendía M., García-Ruiz E., Bazo A., Bolea-Fernandez E., Vanhaecke F. (2022). Living in a Transient World: ICP-MS Reinvented via
Time-Resolved Analysis for Monitoring Single Events. Chem. Sci..

[25] Degueldre C., Favarger P.-Y. (2003). Colloid Analysis by Single Particle Inductively Coupled
Plasma-Mass Spectroscopy: A Feasibility Study. Colloids Surf., A.

[26] Pace H. E., Rogers N. J., Jarolimek C., Coleman V. A., Higgins C. P., Ranville J. F. (2011). Determining Transport
Efficiency for the Purpose of
Counting and Sizing Nanoparticles via Single Particle Inductively
Coupled Plasma Mass Spectrometry. Anal. Chem..

[27] Laborda F., Jiménez-Lamana J., Bolea E., Castillo J. R. (2013). Critical
Considerations for the Determination of Nanoparticle Number Concentrations,
Size and Number Size Distributions by Single Particle ICP-MS. J. Anal. At. Spectrom..

[28] Bazo A., Bolea-Fernandez E., Rua-Ibarz A., Aramendía M., Resano M. (2024). Intensity- and Time-Based Strategies for Micro/Nano-Sizing
via Single-Particle ICP-Mass Spectrometry: A Comparative Assessment
Using Au and SiO_2_ as Model Particles. Anal. Chim. Acta.

[29] Theiner S., Loehr K., Koellensperger G., Mueller L., Jakubowski N. (2020). Single-Cell
Analysis by Use of ICP-MS. J. Anal. At. Spectrom..

[30] Corte-Rodríguez M., Álvarez-Fernández R., García-Cancela P., Montes-Bayón M., Bettmer J. (2020). Single Cell ICP-MS Using on Line
Sample Introduction Systems: Current Developments and Remaining Challenges. Trac-Trends Anal. Chem..

[31] Velimirovic M., Tirez K., Verstraelen S., Frijns E., Remy S., Koppen G., Rotander A., Bolea-Fernandez E., Vanhaecke F. (2021). Mass Spectrometry as a Powerful Analytical
Tool for
the Characterization of Indoor Airborne Microplastics and Nanoplastics. J. Anal. At. Spectrom..

[32] Hua J., Gengsheng J. (2009). Hydrothermal Synthesis and Characterization of Monodisperse
α-Fe_2_O_3_ Nanoparticles. Mater. Lett..

[33] Pereira J. S. F., Álvarez-Fernández García R., Corte-Rodríguez M., Manteca A., Bettmer J., LeBlanc K. L., Mester Z., Montes-Bayón M. (2023). Towards Single
Cell ICP-MS Normalized Quantitative Experiments Using Certified Selenized
Yeast. Talanta.

[34] Bazo A., Bolea-Fernandez E., Rua-Ibarz A., Aramendía M., Resano M. (2025). Ions with Ions, Entities
with Entities: A Proof-of-Concept
Study Using the SELM-1 Yeast Certified Reference Material for Intra-
and Extracellular Se Quantification via Single-Cell ICP-Mass Spectrometry. Anal. Chem..

[35] Bolea-Fernandez E., Leite D., Rua-Ibarz A., Liu T., Woods G., Aramendia M., Resano M., Vanhaecke F. (2019). On the Effect
of Using Collision/Reaction Cell (CRC) Technology in Single-Particle
ICP-Mass Spectrometry (SP-ICP-MS). Anal. Chim.
Acta.

[36] Bolea-Fernandez E., Rua-Ibarz A., Velimirovic M., Tirez K., Vanhaecke F. (2020). Detection
of Microplastics Using Inductively Coupled Plasma-Mass Spectrometry
(ICP-MS) Operated in Single-Event Mode. J. Anal.
At. Spectrom..

[37] Liu T., Bolea-Fernandez E., Mangodt C., De Wever O., Vanhaecke F. (2021). Single-Event
Tandem ICP-Mass Spectrometry for the Quantification of Chemotherapeutic
Drug-Derived Pt and Endogenous Elements in Individual Human Cells. Anal. Chim. Acta.

[38] Agatemor C., Beauchemin D. (2011). Towards the Reduction of Matrix Effects
in Inductively
Coupled Plasma Mass Spectrometry without Compromising Detection Limits:
The Use of Argon-Nitrogen Mixed-Gas Plasma. Spectrochim. Acta, Part B.

[39] Gschwind S., Flamigni L., Koch J., Borovinskaya O., Groh S., Niemax K., Günther D. (2011). Capabilities
of Inductively Coupled Plasma Mass Spectrometry for the Detection
of Nanoparticles Carried by Monodisperse Microdroplets. J. Anal. At. Spectrom..

[40] Olesik J. W., Gray P. J. (2012). Considerations for Measurement of Individual Nanoparticles
or Microparticles by ICP-MS: Determination of the Number of Particles
and the Analyte Mass in Each Particle. J. Anal.
At. Spectrom..

[41] Kálomista I., Kéri A., Ungor D., Csapó E., Dékány I., Prohaska T., Galbács G. (2017). Dimensional
Characterization of Gold Nanorods by Combining Millisecond and Microsecond
Temporal Resolution Single Particle ICP-MS Measurements. J. Anal. At. Spectrom..

[42] Kéri A., Kálomista I., Ungor D., Bélteki Á., Csapó E., Dékány I., Prohaska T., Galbács G. (2018). Determination of the Structure and Composition of Au-Ag
Bimetallic Spherical Nanoparticles Using Single Particle ICP-MS Measurements
Performed with Normal and High Temporal Resolution. Talanta.

[43] Kutralam-Muniasamy G., Shruti V. C., Pérez-Guevara F., Flores J. A. (2024). The Emerging
Field of Inductively Coupled Plasma Mass Spectrometry for (Micro)­Nanoplastic
Analysis: “The 3As” Analysis, Advances, and Applications. Trac-Trends Anal. Chem..

[44] Gonzalez
de Vega R., Goyen S., Lockwood T. E., Doble P. A., Camp E. F., Clases D. (2021). Characterisation of Microplastics
and Unicellular Algae in Seawater by Targeting Carbon via Single Particle
and Single Cell ICP-MS. Anal. Chim. Acta.

[45] Laborda F., Trujillo C., Lobinski R. (2021). Analysis of
Microplastics in Consumer
Products by Single Particle-Inductively Coupled Plasma Mass Spectrometry
Using the Carbon-13 Isotope. Talanta.

[46] Van
Acker T., Rua-Ibarz A., Vanhaecke F., Bolea-Fernandez E. (2023). Laser Ablation for Nondestructive Sampling of Microplastics
in Single-Particle ICP-Mass Spectrometry. Anal.
Chem..

[47] Sandro F., Bodo H., Detlef G. (2025). Quantitative Sizing of Microplastics
up to 20 μm Using ICP-TOFMS. J. Anal.
At. Spectrom..

[48] Kuonen M., Niu G., Hattendorf B., Günther D. (2023). Characterizing a nitrogen microwave
inductively coupled atmospheric-pressure plasma ion source for element
mass spectrometry. J. Anal. At. Spectrom..

