# Supplementary material for: Discrete Entity Analysis via Microwave-Induced Nitrogen Plasma–Mass Spectrometry in Single-Event Mode
Source: Anal Chem. 2025 Oct 14;97(43):24065–72. doi: 10.1021/acs.analchem.5c04341 (PMC12590458; doi:10.1021/acs.analchem.5c04341)
Supplement: Supplementary file 1 [file ac5c04341_si_001.pdf]

Supporting Information

Discrete entity analysis via microwave-induced nitrogen plasma-mass spectrometry in single-event mode

Ana Rua-Ibarz[1], Flávio V. Nakadi[1], Eduardo Bolea-Fernandez[1], Antonio Bazo[1], Beatrice Battistella[2], Anna Matiushkina[2], Ute Resch-Genger[2], Carlos Abad[2]*, Martín Resano[1]*

[1]University of Zaragoza, Department of Analytical Chemistry, Aragon Institute of Engineering Research (I3A), Zaragoza, Spain, 50009

[2]Bundesanstalt für Materialforschung und -prüfung (BAM), Richard-Willstätter-Str. 11, D-12489 Berlin, Germany

*Corresponding authors: Carlos Abad (carlos.abad@bam.de); Martín Resano (mresano@unizar.es)

Table of contents

Materials, synthesis and characterization of α-Fe_2O_3 NPs ………………………………………………… S2

References…… S2

Materials, synthesis and characterization of α-Fe_2O_3 NPs

Materials. Iron (III) chloride hexahydrate (≥99%) was obtained from Chemsolute (Th Geyer, Germany), sodium acetate trihydrate (pharma grade) from PanReac AppliChem, ethylene glycol (≥99%) from Carl Roth GmbH (Germany), and sodium citrate tribasic dihydrate (≥99.5%) from Fluka. All chemicals were used without further purification. All aqueous solutions were prepared with deionized water (0.055 µS·m^{-1}; Milli-Q water, Millipore).

Synthesis of α-Fe_2O_3 NPs and surface modification with citrate. Custom-made hematite (α-Fe_2O_3) nanoparticles with a size of around 65 nm and stabilized by citrate on the particles' surface were synthesized using a slightly modified hydrothermal synthesis approach previously reported,[1] followed by surface functionalization with citrate. Thereby, one solution containing 270.4 mg (1 mmol) of iron(III) chloride hexahydrate in 2 mL of Milli-Q water and a solution of 408.4 mg (3 mmol) of sodium acetate trihydrate dissolved in 6 mL of Milli-Q water were prepared and mixed at room temperature (r.t., T = 23 °C) under continuous stirring. Subsequently, 1 mL (18 mmol) of ethylene glycol were dropwise added under vigorous stirring at a rate of around 5 drops/min. Afterward, 1 mL of Milli-Q water were added, and the reaction mixture was stirred for an additional 30 min to ensure homogeneity. The reaction mixture was then transferred to a sealed autoclave and heated at 180 °C for 48 hours. Then, the autoclave was cooled to r.t. under ambient conditions. The resulting hematite nanoparticles were collected by centrifugation (6000 relative centrifugal force (rcf), 5 min) and washed three times with 15 mL of Milli-Q water. Finally, the hematite nanoparticles were redispersed in 15 mL of Milli-Q water. To enhance the colloidal stability of the α-Fe_2O_3 nanoparticles, a post-synthetic surface modification was performed by mixing the particles with excess sodium citrate overnight. Specifically, 2 mL of the as-synthesized hematite nanoparticle dispersion were combined with 109.2 mg (0.37 mmol) of sodium citrate tribasic dihydrate in 2 mL of Milli-Q water and shaken overnight at r.t. using a vortex laboratory shaker (Vortex Genie 2, Scientific Industries). The citrate-stabilized hematite nanoparticles were then purified by centrifugation (8000 rcf, 5 min) and washed twice with 4 mL of Milli-Q water. Finally, the citrate-modified hematite nanoparticles were redispersed and homogenized in 2 mL of Milli-Q water.

Transmission electron microscopy (TEM). TEM measurements were performed using a Talos F200S microscope (Thermo Fisher Scientific), operating at an accelerating voltage of 200 kV. The samples were drop-cast onto carbon-coated copper grids (Plano GmbH) and dried at r.t. The TEM micrographs were analyzed using the ImageJ software (Version 1.54g), evaluating around 200 particles for α-Fe_2O_3 NPs sample.

Dynamic light scattering (DLS) measurements. DLS measurements were carried out at 25 °C with a Zetasizer Nano ZS (Malvern Panalytical Ltd.), equipped with a 633 nm laser, and using a back scattering detection angle of 173°. Each Fe-based NPs sample was measured in triplicate using standard 1 cm disposable cuvettes (Sarstedt). The hydrodynamic diameter was derived with the cumulant method and calculated for a number-based distribution using refractive indexes of 2.80 and 1.33 for iron oxide and water, respectively, as well as a viscosity of 0.8872 cP for water.

References

1. Hua, J., and Gengsheng, J. Hydrothermal synthesis and characterization of monodisperse α-Fe2O3 nanoparticles. *Materials Letters* **2009**, *63* (30), 2725-2727. DOI: https://doi.org/10.1016/j.matlet.2009.09.054.
